# Within-Person Associations of Accelerometer-Assessed Physical Activity With Time-Varying Determinants in Older Adults: Time-Based Ecological Momentary Assessment Study

**DOI:** 10.2196/44425

**Published:** 2023-11-23

**Authors:** Iris Maes, Lieze Mertens, Louise Poppe, Tomas Vetrovsky, Geert Crombez, Femke De Backere, Ruben Brondeel, Delfien Van Dyck

**Affiliations:** 1 Department of Movement and Sports Sciences Ghent University Ghent Belgium; 2 Department of Public Health and Primary Care Ghent University Ghent Belgium; 3 Fund for Scientific Research Flanders Brussels Belgium; 4 Faculty of Physical Education and Sport Charles University Prague Czech Republic; 5 Department of Experimental-Clinical and Health Psychology Ghent University Ghent Belgium; 6 Department of Information Technology Ghent University Ghent Belgium; 7 Department of Epidemiology of Infectious Diseases Belgian Institute for Health (Sciensano) Brussels Belgium

**Keywords:** ecological momentary assessment, EMA, associations, emotions, physical concerns, intention, self-efficacy, older adult, mobile phone

## Abstract

**Background:**

Despite the availability of physical activity (PA) interventions, many older adults are still not active enough. This might be partially explained by the often-limited effects of PA interventions. In general, health behavior change interventions often do not focus on contextual and time-varying determinants, which may limit their effectiveness. However, before the dynamic tailoring of interventions can be developed, one should know which time-dependent determinants are associated with PA and how strong these associations are.

**Objective:**

The aim of this study was to examine within-person associations between multiple determinants of the capability, opportunity, motivation, and behavior framework assessed using Ecological Momentary Assessment (EMA) and accelerometer-assessed light PA, moderate to vigorous PA, and total PA performed at 15, 30, 60, and 120 minutes after the EMA trigger.

**Methods:**

Observational data were collected from 64 healthy older adults (36/64, 56% men; mean age 72.1, SD 5.6 y). Participants were asked to answer a time-based EMA questionnaire 6 times per day that assessed emotions (ie, relaxation, satisfaction, irritation, and feeling down), the physical complaint fatigue, intention, intention, and self-efficacy. An Axivity AX3 was wrist worn to capture the participants’ PA. Multilevel regression analyses in R were performed to examine these within-person associations.

**Results:**

Irritation, feeling down, intention, and self-efficacy were positively associated with subsequent light PA or moderate to vigorous PA at 15, 30, 60, or 120 minutes after the trigger, whereas relaxation, satisfaction, and fatigue were negatively associated.

**Conclusions:**

Multiple associations were observed in this study. This knowledge in combination with the time dependency of the determinants is valuable information for future interventions so that suggestions to be active can be provided when the older adult is most receptive.

## Introduction

Over the years, the World Health Organization has established and updated physical activity (PA) guidelines. Currently, the recommendation for older adults is to perform at least 150 to 300 minutes of moderate PA, at least 75 to 150 minutes of vigorous PA, or an equivalent combination of both throughout the week [1]. The level of PA typically decreases with age, and this decrease is associated with a decline in functional fitness [2,3], which in turn has negative consequences for healthy aging. Despite the awareness of the PA guidelines and the availability of PA interventions, meeting these guidelines is a challenge for many older adults: they are the least active age group compared with the other age groups [3,4].

Many PA interventions often have limited or only short-term health effects [5,6], which might be partly explained by the fact that the behavioral determinants of PA have been considered relatively stable over time and contexts. In real life, determinants are not stable but vary over time and depend on contextual factors [7]. For example, an individual might have the intention to go for a walk, but during the day, their back starts to hurt, and as a result, their intention to be active changes and they might choose to stay at home and read a book instead. Such contextual and time-varying variables are not usually the focus of PA interventions. Recently, more attention has been drawn to the dynamic aspects of behavioral determinants [8-10]. Current technology (eg, smartphones) provides easy opportunities to monitor individuals more frequently and continuously and consequently could help tailor interventions to the constantly changing determinants of individuals. However, before dynamic tailoring of interventions can be developed, one should know which time- and context-dependent determinants are associated with PA and how strong these associations are. Thus, the most influential determinants can be targeted or incorporated into eHealth and mobile health (mHealth) [11] interventions to promote PA more effectively and achieve long-term health benefits.

Ecological Momentary Assessment (EMA), also known as the Experience Sampling Method or Ambulatory Assessment, is a method with high potential to assess the time- and context-dependent fluctuations in determinants. EMA captures real-time data at multiple points in time in an individual’s natural environment and facilitates the examination of short-term changes in, for example, emotions, temporal dynamics, and the effects of specific contexts [7]. Guidance from theoretical frameworks is needed to decide which determinants to examine [12]. Although many theoretical frameworks can be used as dynamic frameworks to interpret behavioral determinants as time and context dependent, studies are often not designed in this manner. In this study, the capability, opportunity, motivation, and behavior (COM-B) framework was used to identify the behavioral determinants of PA and develop an EMA questionnaire. The COM-B framework consists of 3 constructs: capability, opportunity, and motivation. Capability is the psychological or physical ability of a person to enact the health behavior, which can include physical complaints such as fatigue. Opportunity comprises the physical and social environment that enables behavior, but this component was not assessed in this study. Finally, motivation contains the reflective and automatic mechanisms that activate or inhibit behavior including, for example, emotions, intention, and self-efficacy. Capability and opportunity can both influence motivation. Furthermore, capability, opportunity, and motivation can influence health behavior, but performing a certain behavior (eg, PA) can also alter these 3 constructs [13]. By identifying the time- and context-dependent variations of determinants and by examining their influence on subsequent PA, future eHealth and mHealth interventions such as just-in-time adaptive interventions (JITAIs) can take the individuals’ emotional, cognitive, and physical momentary states into account to determine the “right” time to stimulate individuals to be active [14]. For example, the intervention can be programmed to provide suggestions to be active (eg, take a short walk) through a smartphone app when the intention to be active is high, and there are no physical complaints (eg, absence of pain).

The knowledge of which determinants are influential and how strong their influence is essential to target these dynamic determinants in eHealth and mHealth interventions. In the research field of PA, some research has been conducted to examine the associations of determinants with subsequent PA using EMA, but most studies have been conducted in children [15], adolescents [16,17], and adults [18-21]. To our knowledge, only a few studies have been conducted in older adults [22-24]. These were mainly focused on interrupting subsequent sedentary behavior or studies in which PA was self-reported. For example, intention to stand and move as well as self-efficacy beliefs about one’s ability to stand or move predicted increases in the subsequent time spent upright in the 2 hours following the EMA trigger. Furthermore, intentions to limit time spent sitting as well as self-efficacy beliefs about one’s ability to limit time spent sitting resulted in more time spent upright in the subsequent 2 hours in older adults [22]. Another study found that greater levels of self-reported energy led to more time standing and stepping in the subsequent 15 and 30 minutes after the EMA trigger [23]. However, no associations between positive and negative affect with subsequent standing or stepping were found [23]. Dunton et al [24] found that greater self-efficacy and positive affect predicted higher levels of subsequent self-reported PA, whereas greater negative affect predicted lower levels of subsequent self-reported PA. In the same study, fatigue was unrelated to subsequent PA [24]. Therefore, although a few studies have examined the association of determinants with subsequent standing and stepping or subsequent self-reported PA, research examining the associations of time-dependent determinants with accelerometer-assessed subsequent PA in older adults is lacking.

The aim of this study was to examine predictive within-person associations between multiple determinants of the capability and motivation components of the COM-B framework (ie, emotions, physical complaints, intention, and self-efficacy) that were assessed using time-based EMA and subsequent accelerometer-assessed PA. Specifically, the study examined within-person associations with light PA (LPA), moderate to vigorous PA (MVPA), and total PA (TPA) performed in the 15, 30, 60, and 120 minutes after the EMA trigger. Various time frames were selected to capture the acute temporal nature of the associations and to examine whether the associations differed or changed depending on the time frame. On the basis of the results of previous research in adults [19-21] and previous research on motivators and barriers to PA [25], we hypothesized that the positive emotions, intention, and self-efficacy are positively related to subsequent PA, whereas negative emotions and physical complaints are negatively associated with subsequent PA. By exploring these predictive within-person associations, we aimed to identify important time-dependent determinants that should be targeted in future JITAIs to more effectively promote PA in older adults. The target population of this study was older adults. As the importance of psychosocial factors (eg, motivation) differs depending on age [26-28], it might be possible that different psychosocial determinants concerning PA are important for different age groups. Consequently, the associations between these individual-level determinants may differ between age groups. In addition, the activity radius of older adults is limited compared with other age groups; therefore, they presumably have a limited number of contexts in which they interact. Furthermore, older adults often have a more flexible day schedule (because most of them are retired) and they are a crucial age group to target in health interventions during this time of increased life expectancy and “healthy aging.” All these aspects make them a very appropriate target group to receive “in-the-moment” interventions.

## Methods

### Participants

This study was conducted in Belgium. Healthy older adults (aged ≥65 y) were recruited between November 2019 and March 2020 using convenience sampling (ie, flyers on social media and contacting associations for older adults). Self-reported exclusion criteria were (1) impaired cognition (ie, diagnosed with dementia and Alzheimer or other cognitive diseases), (2) severe impairment of vision and hearing, (3) inability to walk 100 m and stand or sit independently, (4) impairment of fine motor skills, and (5) insufficient knowledge of the Dutch language.

### Procedures

The Checklist for Reporting EMA Studies reporting guidelines proposed by Liao et al [29] were used to describe this EMA study. Participants were visited twice at home. During the first visit, informed consent was signed; sociodemographics (ie, sex; age; BMI; educational level, ie, nontertiary education—none, primary education, or secondary education—and tertiary education—higher education or university education), main occupation before retirement, marital status, having children and grandchildren were collected through a paper-based questionnaire; and instructions for the measurement period were given. In addition, the EMA app was installed on the participants’ smartphones, followed by a brief training on how to use it (ie, opening the app and answering the EMA questionnaires) by providing printed screenshots of the app. The visit was followed by one monitoring period of 7 consecutive days, consisting of 5 weekdays and 2 weekend days, although not all participants started the measurement period on the same day of the week. During this measurement period, participants were asked to answer 6 EMA questionnaires per day using a smartphone app and to wear an Axivity AX3 accelerometer to monitor their PA. After these 7 days, a second home visit was performed, during which the measurement materials were reassembled.

### Ethics Approval

Ethics approval was obtained from the Ghent University Hospital Ethics Committee before the start of the study (2019/0192).

### EMA Protocol

The Smartphone EMA^3^ (SEMA^3^) app is a suite of software for intensive longitudinal survey research that can be used on iOS and Android smartphones [30]. The SEMA^3^ app triggered 6 time-based EMA questionnaires per day, between 9 AM and 10 PM, for 7 consecutive days (ie, the participants were required to answer 42 questionnaires in total). Each EMA questionnaire was randomly triggered within a predefined time frame of 1 hour (eg, the time frame from 9 AM to 10 AM, in which the EMA trigger randomly appeared at 9:38 AM). In total, 6 time frames were predefined per day. If the participants did not respond to the initial trigger, 2 reminders were given after approximately 5 minutes and 10 minutes; 20 minutes after the initial trigger, the questionnaire was unavailable until the next scheduled trigger. Participants were asked to use their own smartphone during the measurement period (the lowest acceptable operating systems were Android 5.0 and iOS 12.4), but participants who did not own a smartphone were provided with a Wiko Lenny 3 smartphone (Android 6.0).

### EMA Questionnaire

The COM-B framework was used as a guiding framework to compile the first version of the questionnaire from items previously used in research [31-33]. Experts in psychology, health sciences, and EMA were involved at multiple stages in the development of the EMA questionnaire. Subsequently, cognitive interviews were conducted with 10 older adults, which led to further adjustments of the questionnaire, mainly concerning the comprehensibility of the items. The final version of the EMA questionnaire assessed the following components in this fixed order: emotions, physical complaints, and the constructs’ intention and self-efficacy. However, the order of the questions within the components’ emotions and physical complaints was randomized and therefore could change over the EMA triggers. In total, the EMA questionnaire consisted of 18 items [34]. However, based on the results of a previous analysis of the same EMA data [34], only those determinants with more within-subject variation (>50%) than between-subject variation were selected for this study. Consequently, only 7 items of the EMA questionnaire were analyzed in this study: relaxation, satisfaction, irritation, feeling down, fatigue, intention, and self-efficacy. The items for the emotions relaxation, satisfaction, irritation, and feeling down were originally developed by the research group of Philippe Delespaul at the University of Maastricht [31]. The item for physical complaint fatigue was selected from the validated Patient Health Questionnaire-15 [32]. Finally, the items assessing intention and self-efficacy toward PA were based on items that are frequently used in our research group [33] but were specifically adapted by the authors for this EMA study. All items were assessed on a 7-point Likert scale and were presented in Dutch (their English translation is available in Multimedia Appendix 1).

### Axivity AX3 Accelerometer

To capture participants’ PA, all participants wore an Axivity AX3 accelerometer on their nondominant wrist during the same 7 consecutive days as the time-based EMA. The Axivity AX3 accelerometer is a reliable and valid device for measuring PA [35]. Participants were instructed to wear the accelerometer during the whole day and night and to remove the accelerometer only during water-based activities. Data were extracted using the OMGUI software (Open Movement) [36] and then processed in R (version 4.0.1; R Foundation for Statistical Computing) [37]. The raw data downloaded from Axivity AX3 were first reduced by averaging the Euclidean norm minus one values (in milligravitational units, ie, mg) over 1-second epochs and then 1-minute epochs, after which cut points for older adults by Sanders et al [38] were applied to categorize individual minutes as sedentary (≤57 mg), LPA (57-104 mg), or MVPA (≥104 mg). The total number of LPA and MVPA minutes was calculated for the time frames of 15, 30, 60, and 120 minutes after the trigger. In addition, the total number of TPA minutes was obtained for these time frames by summing LPA and MVPA minutes.

### Analyses

Multilevel regression analyses were performed using the lme4 package [39] in R [37]. Residuals were plotted and visually inspected to check for linearity and normality assumptions. Because the PA data in the 4 time frames after the EMA trigger (ie, 15, 30, 60, and 120 min) included an excessive number of “true” null values (ie, not all participants were physically active during all time frames), Hurdle models [40,41] were fitted. The Hurdle models consist of 2 parts: a logistic regression model, estimating the odds of engaging in PA after the EMA trigger, and a linear model, estimating associations with the amount of PA among those who performed at least some PA at 15, 30, 60, and 120 minutes after the EMA trigger. First, the logistic regression model was applied, for which all PA variables were recoded into dichotomous variables (ie, 0 vs at least 1 min of LPA, MVPA, and TPA, respectively). For each determinant, a separate model was created with each PA variable (ie, LPA, MVPA, and TPA in the 4 different time frames) as an outcome variable, which led to 84 models in total (ie, 7 determinants × 3 PA outcome variables × 4 time frames). Further model assumptions were visually checked (ie, outliers and influential observations). Second, to construct the linear model, 3 different models were applied to the original PA values for each determinant separately to check which model best fitted the data (ie, Gaussian, Poisson, and negative binomial). The models were fitted separately for each determinant and each PA outcome. Of these 3 models, the model with the lowest Akaike information criterion value indicating a better model fit was chosen. The Akaike information criterion was, in all cases, the lowest for the negative binomial model; therefore, the negative binomial was applied in all cases. The models were fitted for each determinant as between-subject (ie, mean of the variable at the subject level) as well as within-subject (ie, individuals’ score minus their mean score), but only the within-subject associations were considered in this study. In addition, for the linear models, further model assumptions were visually checked (ie, homoscedasticity, outliers, and influential observations). The level of significance was set at α<.05. To limit the number of results for this study, all results for TPA can be found in Multimedia Appendices 2-4.

## Results

### Descriptive Statistics

In total, 67 older adults participated in this study. All participants completed the measurement period of 7 days. To be included in the analysis, participants had to respond to at least one-third of all triggers (ie, 14 out of 42). As a result, data from 3 participants were excluded, and 64 participants were included in the analysis. Their descriptive statistics are presented in Table 1. In total, 11 participants used a Wiko Lenny 3 smartphone because they did not have a smartphone of their own.

A total of 2690 triggers were sent during the study, of which 30 were not delivered as intended because of technical issues (ie, more than 6 triggers a day were sent or triggers were sent outside the predefined time frames, eg, during the night) and were excluded from the analysis. Of the remaining 2660 triggers, 2057 EMA questionnaires were completed (response rate of 2057/2660, 77.33% and a mean of 32.1 completed questionnaires per participant). The median response latency was 0.00 (quarter 1=0.00, quarter 3=4.00) minutes. The median time needed to complete the EMA questionnaire was 1.79 (quarter 1=1.43, quarter 3=2.31) minutes. Descriptive statistics for LPA and MVPA are presented in Table 2, and those for the EMA items are presented in Table 3.

**Table 1:** Descriptive statistics of the study sample (N=64).

Demographics		Values
Sex (male), n (%)		36 (56)
Age (y), mean (SD; range)		72.1 (5.9; 65-86)
BMI (kg/m^2^), mean (SD; range)		25.6 (4.1; 15.2-36.0)
Nontertiary education, n (%)		37 (58)
**Main occupation before retirement, n (%)**		
	Household	3 (5)
	Blue collar worker^a^	23 (36)
	White collar worker^b^	34 (53)
	Other	4 (6)
**Marital status, n (%)**		
	Single	2 (3)
	Married or living together	54 (84)
	Divorced	2 (3)
	Widow or widower	6 (9)

^a^Self-employed and worker.

^b^Employee, education, executives, free professions, and officer.

**Table 2:** Descriptive statistics of the physical activity (PA) data for light PA (LPA) and moderate to vigorous PA (MVPA) in the 15, 30, 60, and 120 minutes after the trigger.

			Active min, mean (SD)		Active min, median (range)		Quarter 1-quarter 3		Percentage of people who did not perform any PA
**LPA (min after trigger)**									
	15	1.8 (2.6)		0.0 (0.0-15.0)		0.0-3.0		51.6	
	30	3.4 (4.5)		2.0 (0.0-27.0)		0.0-5.0		37.5	
	60	6.8 (7.9)		4.0 (0.0-55.0)		1.0-10.0		21.9	
	120	13.0 (13.3)		9.0 (0.0-88.0)		3.0-19.0		9.8	
**MVPA (min after trigger)**									
	15	0.6 (1.8)		0.0 (0.0-15.0)		0.0-0.0		80.8	
	30	1.3 (3.3)		0.0 (0.0-27.0)		0.0-1.0		70.8	
	60	2.4 (5.6)		0.0 (0.0-53.0)		0.0-2.0		58.3	
	120	4.5 (9.3)		1.0 (0.0-95.0)		0.0-4.0		43.5	

**Table 3:** Descriptive statistics of the Ecological Momentary Assessment items^a^.

Item	Score, mean (SD)	Score, median	Quarter 1-quarter 3
Relaxation	4.7 (1.5)	5.0	4.0-5.0
Satisfaction	4.8 (1.4)	5.0	4.0-6.0
Irritation	1.5 (1.0)	1.0	1.0-1.0
Feeling down	1.3 (0.8)	1.0	1.0-1.0
Fatigue	1.9 (1.1)	1.0	1.0-3.0
Intention	3.7 (2.1)	3.0	2.0-5.0
Self-efficacy	4.2 (2.1)	4.0	3.0-6.0

^a^All items had a minimum of 1 and a maximum of 7 (range 1-7), that is, for example, 1=not at all and 7=very relaxed.

### Within-Person Associations With Subsequent PA

#### Overview

In Table 4, detailed results for the logistic models and negative binomial models are presented. A visual summary of the direction of the associations of all determinants with LPA and MVPA in the 15, 30, 60, and 120 minutes after the EMA trigger is provided in Multimedia Appendix 5. A summary of the significant associations found in the logistic and negative binomial models is provided in subsequent sections.

#### Relaxation

A 1-unit increase in “relaxation” was associated with 9%, 10%, 11%, and 24% lower odds of performing any LPA in the 15 minutes (*P*=.03), 30 minutes (*P*=.02), 60 minutes (*P*=.04), and 120 minutes (*P*=.002) after the trigger, respectively. In participants who performed any LPA, a 1-unit increase in “relaxation” was associated with 5% and 6% fewer minutes of LPA, both in the 60 minutes (*P*=.006) and 120 minutes (*P*<.001) after the trigger, respectively.

A 1-unit increase in “relaxation” was associated with 9% lower odds of performing any MVPA, both in the 60 minutes (*P*=.03) and 120 minutes (*P*=.03) after the trigger. In participants who performed any MVPA, a 1-unit increase in “relaxation” was associated with 6% fewer minutes of MVPA in the 60 minutes (*P*=.05) after the trigger.

#### Satisfaction

A 1-unit increase in “satisfaction” was associated with 9% lower odds of performing any LPA in the 15 minutes (*P*=.04) after the trigger. In participants who performed any LPA, a 1-unit increase in “satisfaction” was associated with 4% and 5% fewer minutes of LPA in the 60 minutes (*P*=.02) and 120 minutes (*P*=.002) after the trigger, respectively.

No significant associations were found between satisfaction and MVPA in the negative binomial model.

#### Irritation

A 1-unit increase in “irritation” was associated with 13% and 28% higher odds of performing any LPA in the 30 minutes (*P*=.04) and 120 minutes (*P*=.03) after the trigger, respectively. In participants who performed any LPA, a 1-unit increase in “irritation” was associated with 6% more minutes of LPA in the 120 minutes (*P*=.01) after the trigger.

A 1-unit increase in “irritation” was associated with 16% higher odds of performing any MVPA within the 120 minutes (*P*=.01) after the trigger. No significant associations were found between irritation and MVPA in the negative binomial model.

#### Feeling Down

A 1-unit increase in “feeling down” was associated with 23%, 19%, 22%, and 45% higher odds of performing any LPA in the 15 minutes (*P*=.003), 30 minutes (*P*=.03), 60 minutes (*P*=.03), and 120 minutes (*P*=.02) after the trigger, respectively. In participants who performed any LPA, a 1-unit increase in “feeling down” was associated with 7% more minutes of LPA in the 120 minutes (*P*=.02) after the trigger.

No significant associations were found between feeling down and MVPA in the logistic model nor in the negative binomial model.

#### Fatigue

A 1-unit increase in “fatigue” was associated with 18% and 26% lower odds of performing any LPA in the 60 minutes (*P*=.002) and 120 minutes (*P*=.001) after the trigger, respectively. In participants who performed any LPA, a 1-unit increase in “fatigue” was associated with 5%, 7%, 8%, and 12% fewer minutes of LPA in the 15 minutes (*P*=.06), 30 minutes (*P*=.006), 60 minutes (*P*<.001), and 120 minutes (*P*<.001) after the trigger, respectively.

A 1-unit increase in “fatigue” was associated with 18% and 21% lower odds of performing any MVPA in the 60 minutes (*P*<.001) and 120 minutes (*P*<.001) after the trigger, respectively. In participants who performed any MVPA, a 1-unit increase in “fatigue” was associated with 8% and 12% fewer minutes of MVPA in the 60 minutes (*P*=.05) and 120 minutes (*P*<.001) after the trigger, respectively.

#### Intention

A 1-unit increase in “intention” was associated with 33%, 40%, 45%, and 46% higher odds of performing any LPA in the 15 minutes (*P*<.001), 30 minutes (*P*<.001), 60 minutes (*P*<.001), and 120 minutes (*P*<.001) after the trigger, respectively. In participants who performed any LPA, a 1-unit increase in “intention” was associated with 8%, 12%, 19%, and 24% more minutes of LPA in the 15 minutes (*P*<.001), 30 minutes (*P*<.001), 60 minutes (*P*<.001), and 120 minutes (*P*<.001) after the trigger, respectively.

A 1-unit increase in “intention” was associated with 38%, 42%, 45%, and 48% higher odds of performing any MVPA in the 15 minutes (*P*<.001), 30 minutes (*P*<.001), 60 minutes (*P*<.001), and 120 minutes (*P*<.001) after the trigger, respectively. In participants who performed any MVPA, a 1-unit increase in “intention” was associated with 12%, 14%, 22%, and 28% more minutes of MVPA in the 15 minutes (*P*<.001), 30 minutes (*P*<.001), 60 minutes (*P*<.001), and 120 minutes (*P*<.001) after the trigger, respectively.

**Table 4:** Outcomes of the logistic models and negative binomial models for light physical activity (LPA) and moderate to vigorous physical activity (MVPA) in the 15, 30, 60, and 120 minutes after the trigger.

				Logistic model				Negative binomial		
				OR^a^ (95% CI)		*P* value		expB^b^ (95% CI)		*P* value
**Relaxation**										
	**LPA (min after trigger)**									
		15	0.91 (0.84-0.99)		.03		0.99 (0.95-1.03)		.49	
		30	0.90 (0.82-0.98)		.02		0.98 (0.95-1.02)		.32	
		60	0.89 (0.80-0.99)		.04		0.95 (0.92-0.99)		.006	
		120	0.76 (0.64-0.90)		.002		0.94 (0.90-0.97)		<.001	
	**MVPA (min after trigger)**									
		15	0.91 (0.83-1.01)		.09		1.01 (0.94-1.08)		.85	
		30	0.93 (0.85-1.02)		.11		0.95 (0.89-1.01)		.11	
		60	0.91 (0.84-0.99)		.03		0.94 (0.89-1.00)		.05	
		120	0.91 (0.83-0.99)		.03		0.95 (0.91-1.00)		.06	
**Satisfaction**										
	**LPA (min after trigger)**									
		15	0.91 (0.83-1.00)		.04		0.99 (0.95-1.03)		.64	
		30	0.91 (0.83-1.00)		.06		0.97 (0.93-1.01)		.16	
		60	0.94 (0.84-1.05)		.25		0.96 (0.92-0.99)		.02	
		120	0.88 (0.75-1.04)		.14		0.95 (0.91-0.98)		.002	
	**MVPA (min after trigger)**									
		15	1.00 (0.90-1.12)		.97		0.99 (0.91-1.07)		.79	
		30	1.00 (0.91-1.10)		.99		0.99 (0.93-1.06)		.85	
		60	0.96 (0.88-1.06)		.42		1.03 (0.96-1.09)		.42	
		120	0.94 (0.86-1.03)		.21		1.02 (0.97-1.08)		.49	
**Irritation**										
	**LPA (min after trigger)**									
		15	1.01 (0.91-1.12)		.87		0.99 (0.94-1.04)		.67	
		30	1.13 (1.01-1.28)		.04		0.99 (0.94-1.04)		.66	
		60	1.14 (0.99-1.31)		.06		1.03 (0.98-1.08)		.27	
		120	1.28 (1.03-1.58)		.03		1.06 (1.01-1.11)		.01	
	**MVPA (min after trigger)**									
		15	1.01 (0.88-1.16)		.88		1.01 (0.88-1.16)		.52	
		30	1.01 (0.90-1.14)		.83		0.95 (0.87-1.04)		.28	
		60	1.06 (0.95-1.19)		.30		0.97 (0.90-1.06)		.53	
		120	1.16 (1.03-1.31)		.01		0.98 (0.91-1.05)		.55	
**Feeling down**										
	**LPA (min after trigger)**									
		15	1.23 (1.07-1.42)		.003		0.99 (0.93-1.05)		.66	
		30	1.19 (1.02-1.38)		.03		1.05 (0.99-1.11)		.12	
		60	1.22 (1.02-1.47)		.03		1.05 (0.99-1.11)		.11	
		120	1.45 (1.06-1.99)		.02		1.07 (1.01-1.12)		.02	
	**MVPA (min after trigger)**									
		15	0.96 (0.80-1.15)		.66		0.94 (0.82-1.08)		.36	
		30	0.94 (0.81-1.10)		.46		0.98 (0.88-1.10)		.79	
		60	1.00 (0.87-1.16)		.96		1.02 (0.91-1.13)		.77	
		120	1.07 (0.92-1.24)		.36		1.01 (0.92-1.09)		.89	
**Fatigue**										
	**LPA (min after trigger)**									
		15	0.94 (0.85-1.05)		.29		0.95 (0.90-1.00)		.06	
		30	0.92 (0.83-1.03)		.15		0.93 (0.89-0.98)		.006	
		60	0.82 (0.72-0.93)		.002		0.92 (0.87-0.96)		<.001	
		120	0.74 (0.61-0.89)		.001		0.88 (0.85-0.92)		<.001	
	**MVPA (min after trigger)**									
		15	0.88 (0.76-1.01)		.07		1.03 (0.93-1.14)		.58	
		30	0.93 (0.82-1.04)		.21		0.97 (0.89-1.06)		.51	
		60	0.82 (0.73-0.92)		<.001		0.92 (0.85-1.00)		.05	
		120	0.79 (0.71-0.89)		<.001		0.88 (0.82-0.94)		<.001	
**Intention**										
	**LPA (min after trigger)**									
		15	1.33 (1.26-1.41)		<.001		1.08 (1.05-1.10)		<.001	
		30	1.40 (1.32-1.50)		<.001		1.12 (1.10-1.15)		<.001	
		60	1.45 (1.34-1.57)		<.001		1.19 (1.16-1.22)		<.001	
		120	1.46 (1.29-1.66)		<.001		1.24 (1.22-1.27)		<.001	
	**MVPA (min after trigger)**									
		15	1.38 (1.28-1.48)		<.001		1.12 (1.07-1.17)		<.001	
		30	1.42 (1.31-1.54)		<.001		1.14 (1.09-1.19)		<.001	
		60	1.45 (1.36-1.55)		<.001		1.22 (1.17-1.27)		<.001	
		120	1.48 (1.39-1.58)		<.001		1.28 (1.23-1.32)		<.001	
**Self-efficacy**										
	**LPA (min after trigger)**									
		15	1.26 (1.19-1.35)		<.001		1.05 (1.02-1.08)		<.001	
		30	1.32 (1.23-1.41)		<.001		1.09 (1.06-1.12)		<.001	
		60	1.37 (1.26-1.49)		<.001		1.15 (1.12-1.18)		<.001	
		120	1.37 (1.21-1.56)		<.001		1.18 (1.15-1.21)		<.001	
	**MVPA (min after trigger)**									
		15	1.30 (1.21-1.41)		<.001		1.10 (1.05-1.15)		<.001	
		30	1.36 (1.27-1.46)		<.001		1.15 (1.10-1.21)		<.001	
		60	1.39 (1.30-1.49)		<.001		1.24 (1.19-1.29)		<.001	
		120	1.39 (1.30-1.49)		<.001		1.21 (1.16-1.26)		<.001	

^a^OR: odds ratio.

^b^expB: beta exponent.

#### Self-Efficacy

A 1-unit increase in “self-efficacy” was associated with 26%, 32%, 37%, and 37% higher odds of performing any LPA in the 15 minutes (*P*<.001), 30 minutes (*P*<.001), 60 minutes (*P*<.001), and 120 minutes (*P*<.001) after the trigger, respectively. In participants who performed any LPA, a 1-unit increase in “self-efficacy” was associated with 5%, 9%, 15%, and 18% more minutes of LPA in the 15 minutes (*P*<.001), 30 minutes (*P*<.001), 60 minutes (*P*<.001), and 120 minutes (*P*<.001) after the trigger, respectively.

A 1-unit increase in “self-efficacy” was associated with 30%, 36%, 39%, and 39% higher odds of performing any MVPA in the 15 minutes (*P*<.001), 30 minutes (*P*<.001), 60 minutes (*P*<.001), and 120 minutes (*P*<.001) after the trigger, respectively. In participants who performed any MVPA, a 1-unit increase in “self-efficacy” was associated with 10%, 15%, 24%, and 21% more minutes of MVPA in the 15 minutes (*P*<.001), 30 minutes (*P*<.001), 60 minutes (*P*<.001), and 120 minutes (*P*<.001) after the trigger, respectively.

## Discussion

### Principal Findings

This study examined the within-person associations of multiple determinants of the capability and motivation component of the COM-B framework with subsequent LPA, MVPA, and TPA. Multiple associations with LPA and MVPA in the 15, 30, 60, and 120 minutes after the trigger were found: irritation, feeling down, intention, and self-efficacy were positively associated with subsequent PA, whereas relaxation, satisfaction, and fatigue were negatively associated with subsequent PA. All results for TPA can be found in Multimedia Appendices 2-4.

In line with our hypothesis, intention and self-efficacy were positively associated with subsequent PA, and fatigue was negatively associated with subsequent PA.

Higher levels of intention or self-efficacy were associated with higher odds of performing subsequent PA or with more minutes of subsequent PA. This finding is in line with previous EMA research in adults and older adults [20,22,24]. In adults, it was found that intentions and self-efficacy positively predicted subsequent accelerometer-assessed MVPA in the 2 hours after the EMA trigger [20]. In older adults, greater levels of self-efficacy predicted higher levels of subjectively measured subsequent MVPA in the 4 hours after the EMA trigger [24]. Intention and self-efficacy were also found to predict increases in the subsequent time spent upright in older adults [22]. Multiple theoretical frameworks [42-44] stress the importance of intention and self-efficacy as a gateway to or as a condition for behavior change, and therefore, positive associations between these constructs and subsequent PA seem natural. However, these theories often do not assess these concepts as dynamic, whereas in a previous study using the same data [34], we found that these are time dependent. Thus, the novelty observed in this study is that these generic associations among intention, self-efficacy, and PA also occur in shorter time frames.

Furthermore, in this study, a negative association was found between the physical complaint fatigue and subsequent LPA in the 15, 30, 60, and 120 minutes after the trigger and subsequent MVPA in the 60 and 120 minutes after the trigger. This means that a higher level of fatigue resulted in lower odds of being physically active and in fewer minutes of subsequent PA. In previous research, fatigue was often mentioned as a barrier to performing PA [25], which consequently might lead to less subsequent PA. However, Dunton et al [24] and Liao et al [19] observed that fatigue was unrelated to subsequent accelerometer-assessed LPA in the 15 and 30 minutes after the EMA trigger in adults [19] and unrelated to subjectively measured subsequent MVPA in the 4 hours after the EMA trigger in older adults [24]. In contrast to the study by Liao et al [19], in this study, significant negative associations of fatigue with LPA were found in all time frames. Although Liao et al [19] did not examine longer time frames, differences in the associations detected 15 and 30 minutes after the trigger might be explained by differences in participants (ie, low-active adults in the study by Liao et al [19]). Differences in results with the study by Dunton et al [24] might be explained by the large time frame and subjective assessment of subsequent PA.

Some of the associations found in this study were unexpected and in contrast with our hypothesis. Irritation and feeling down were seen as negative emotions and were expected to be negatively associated with subsequent PA. However, in this study, the opposite was observed, that is, higher levels of irritation and feeling down resulted in higher odds of being physically active or in more minutes of subsequent PA. Previous research found that higher negative affect (ie, measured as emotionally upset, annoyed, angry, sad, or depressed [45] and as anxious, stressed, depressed, and angry [19]) was associated with lower levels of MVPA in subsequent time frames in older adults [24] and in adults [19]. However, in some other studies, negative affect was positively associated with LPA in the 4 hours after the trigger in older adults [24] and with bodily movement in 15- and 30-minute time frames in adults aged 18 to 73 years [46]. Engaging in PA can improve mental health [1,47-50]; therefore, PA might be used as a coping strategy to counter or reduce the negative emotions of irritation and feeling down to improve affect [51]. In a previous study using the same data [34], we found that for both irritation and feeling down, participants reported in more than 90% of their answers that they were not feeling irritated or feeling down at all, which might have caused a floor effect and influenced the associations identified here. Finally, in this study, higher levels of relaxation and satisfaction resulted in lower subsequent PA. Although relaxation and satisfaction are positive emotions and therefore positive associations with PA were expected, the association with PA might be different than in the case of other positive emotions (eg, cheerfulness). It is possible that older adults who felt relaxed or satisfied did not wish to be active but rather further enjoyed their state of relaxation or satisfaction. However, this is merely an assumption and should be examined further.

In this study, associations were found in different or multiple time frames depending on the determinant that was examined. In light of JITAIs, insight into the importance of determinants in specific time frames is crucial to achieving the greatest behavioral change. For example, when associations are found in the 30 and 60 minutes after the trigger, it might be interesting to examine whether suggestions that encourage the older adult to be active in the next hour instead of “right now” might lead to an increase in their subsequent PA or to examine whether suggestions to be active can be sent after 30 to 60 minutes (after the assessment of the determinant) and in this way increase the older adults’ PA. Both can be examined by using multiple mini interventions. Intention and self-efficacy were positively associated with subsequent PA over all time frames, which shows the importance of these constructs in relation to PA in both short and long time frames. When intention and self-efficacy are already high, it might be sufficient for JITAIs to provide an activity suggestion to increase PA levels. In contrast, when intention and self-efficacy are low, it might be an opportunity for JITAIs to offer behavior change techniques aiming to increase intention and self-efficacy and consequently increase PA. For example, previous research showed that the behavior change technique “provide information on the consequences of behavior in general” is associated with positive changes in intention [52] and that “action planning” was associated with higher self-efficacy [53].

Another observation that could be drawn from the results of this study is that, for some determinants, stronger associations were found with LPA, whereas other determinants were associated with MVPA. For example, emotional satisfaction and feeling down are mainly important for LPA, whereas fatigue is also associated with MVPA. However, these findings are not surprising because they concern different behaviors (ie, examples of LPA are walking and climbing the stairs, while running and cycling are classified as MVPA); therefore, different determinants might be important. Nevertheless, these are important findings for the development of tailored interventions, as different individual determinants may be important for different types of PA that older adults wish to increase. Specifically, suggestions can be tailored according to the behavior of interest, for example, in the case of LPA, providing suggestions to be active when the older adult feels down, and in the case of MVPA, avoiding giving suggestions to be active when the older adult feels fatigued. Intention and self-efficacy showed positive associations with both LPA and MVPA, which provides interesting information for future PA interventions, because both LPA and MVPA can be enhanced in older adults by targeting both determinants.

A previous study using the same data [34] found that multiple individual-level determinants (eg, satisfaction, intention, self-efficacy, and fatigue) are time dependent and therefore can vary within subjects within days. These time-dependent fluctuations are important to keep in mind for the personalization of JITAIs, so that the moment to provide suggestions to be active can be adjusted to the emotional, cognitive, and physical state of the individual. By regularly assessing the time-dependent determinants (eg, multiple times per day) in JITAIs, individually tailored suggestions to improve PA can be provided. Furthermore, it was found that the variation between days was limited in older adults [34]. Therefore, it might be possible to monitor older adults for just a few days before introducing a JITAI to capture the most receptive moments without overburdening them with an extensive monitoring period (eg, 2 weeks). This information, combined with the identified within-person associations found in this study, provides useful information for the development of future JITAIs.

### Strengths and Limitations

A first strength of the study was the repeated assessment throughout the day using EMA. The use of EMA reduces recall biases by capturing present experiences rather than beliefs or ratings based on memory. Furthermore, the assessment occurs in the individuals’ natural environments and social contexts, which increases ecological validity. Finally, fine-grained information was provided using multiple repeated assessments over time. A second strength is that this study fills an important gap in the literature, as previous research on the associations of determinants with subsequent PA is limited, especially in older adults. Third, participants’ PA was accelerometer-assessed using Axivity AX3 accelerometers rather than using self-reported measures.

This study had some limitations. The first limitation is the limited generalizability of the study results, as nonprobability sampling was used to recruit older adults. Therefore, the findings of this study might be specific to the study sample used in this study. It is recommended that future studies use a more random sampling approach to obtain a more heterogeneous study sample and generalize the study results to a wider population of older adults. Second, in this study, PA was considered an outcome variable, with determinants as predictors. However, examining accelerometer-assessed PA as a predictor and the determinants as outcome variables in an EMA study might contribute to a better understanding of the influence of PA on subsequent emotions, physical complaints, and intention and self-efficacy. Third, it is possible that in some cases there was less than 2 hours between 2 triggers, which might have caused some overlap and influenced the associations found in the 120 minutes time frames. This aspect should be considered in future studies. Fourth, although most items of the EMA questionnaire (ie, 4 out of 7) were specifically developed for EMA research, psychometric information was unavailable. It is recommended that future EMA studies use items that are validated for EMA or conduct a validation study before the start of the study. Fifth, we did not consider possible confounders, such as preceding PA, emotions, or other unmeasured variables (eg, sleep). Possible confounders, especially preceding PA, should be considered in future EMA studies. To do so, we first need to explore which time frame of the preceding PA is best to use, since many different time frames can be considered (eg, 15 min, 30 min, and 60 min). However, this exploration was beyond the scope of this study. Sixth, although wrist-worn devices are a common wearing position for the assessment of PA, it might still be possible that there is an overestimation of PA. However, the possibility of an overestimation of PA is limited because the validated cutoff points [38] were used in this study. Although a different accelerometer and different sampling rates were used in the study of Sanders et al [38], the equivalence of the key PA outcomes for GENEActiv and Axivity accelerometers has been proven in previous research [54].

### Conclusions

Overall, this study showed that the emotions irritation and feeling down and the constructs intention and self-efficacy were positively associated with subsequent PA in older adults, whereas the emotions relaxation and satisfaction and physical complaint fatigue were negatively associated with subsequent PA. Using EMA, this study yielded new knowledge about these associations and the time dependency of the determinants, which is valuable for future interventions. By monitoring older adults for a few days, the most receptive moments for triggering them to be more active can be captured, and this information can be used in JITAIs to provide individual tailoring and promote PA more effectively.

## Appendix

Multimedia Appendix 1 Ecological Momentary Assessment questionnaire translated in English.

Multimedia Appendix 2 Descriptive statistics of the total physical activity data.

Multimedia Appendix 3 Outcomes of the logistic models and negative binomial models for total physical activity.

Multimedia Appendix 4 Overview of the associations of the determinants with total physical activity in the 15, 30, 60, and 120 minutes after the trigger.

Multimedia Appendix 5 Overview of the associations of the determinants with light physical activity moderate to vigorous physical activity in the 15, 30, 60 and 120 minutes after.

## Abbreviations

COM-B: capability, opportunity, motivation, and behavior
EMA: Ecological Momentary Assessment
JITAI: just-in-time adaptive intervention
LPA: light physical activity
mHealth: mobile health
MVPA: moderate to vigorous physical activity
PA: physical activity
SEMA3: Smartphone Ecological Momentary Assessment3
TPA: total physical activity
