# Supplementary material for: Within-Person Associations of Accelerometer-Assessed Physical Activity With Time-Varying Determinants in Older Adults: Time-Based Ecological Momentary Assessment Study
Source: JMIR Aging. 2023 Nov 23;6:e44425. doi: 10.2196/44425 (PMC10704312; doi:10.2196/44425)
Supplement: Multimedia Appendix 1 [file aging_v6i1e44425_app1.docx]

## Appendix 1

### EMA questionnaire translated in English

1. How **cheerful** were you just before you received the trigger?

| Not cheerful at all |  | A little cheerful |  | Quite cheerful |  | Very cheerful |
| --- | --- | --- | --- | --- | --- | --- |
| 1 | 2 | 3 | 4 | 5 | 6 | 7 |

1. How **relaxed** were you just before you received the trigger?

| Not relaxed at all |  | A little relaxed |  | Quite relaxed |  | Very relaxed |
| --- | --- | --- | --- | --- | --- | --- |
| 1 | 2 | 3 | 4 | 5 | 6 | 7 |

1. How **enthusiastic** were you just before you received the trigger?

| Not enthusiastic at all |  | A little enthusiastic |  | Quite enthusiastic |  | Very enthusiastic |
| --- | --- | --- | --- | --- | --- | --- |
| 1 | 2 | 3 | 4 | 5 | 6 | 7 |

1. How **satisfied** were you just before you received the trigger?

| Not satisfied at all |  | A little satisfied |  | Quite satisfied |  | Very satisfied |
| --- | --- | --- | --- | --- | --- | --- |
| 1 | 2 | 3 | 4 | 5 | 6 | 7 |

1. How **insecure** were you just before you received the trigger?

| Not insecure at all |  | A little insecure |  | Quite insecure |  | Very insecure |
| --- | --- | --- | --- | --- | --- | --- |
| 1 | 2 | 3 | 4 | 5 | 6 | 7 |

1. How **anxious** were you just before you received the trigger?

| Not anxious at all |  | A little anxious |  | Quite anxious |  | Very anxious |
| --- | --- | --- | --- | --- | --- | --- |
| 1 | 2 | 3 | 4 | 5 | 6 | 7 |

1. How **irritated** were you just before you received the trigger?

| Not irritated at all |  | A little irritated |  | Quite irritated |  | Very irritated |
| --- | --- | --- | --- | --- | --- | --- |
| 1 | 2 | 3 | 4 | 5 | 6 | 7 |

1. How **down** were you **feeling** just before you received the trigger?

| Not feeling down at all |  | Feeling a little down |  | Feeling quite down |  | Feeling very down |
| --- | --- | --- | --- | --- | --- | --- |
| 1 | 2 | 3 | 4 | 5 | 6 | 7 |

1. How **fatigued** did you feel just before you received the trigger?

| Not fatigued at all |  | A little fatigued |  | Quite fatigued |  | Very fatigued |
| --- | --- | --- | --- | --- | --- | --- |
| 1 | 2 | 3 | 4 | 5 | 6 | 7 |

1. How much **pain** did you have just before you received the trigger?

| No pain |  | A little pain |  | Quite some pain |  | A lot of pain |
| --- | --- | --- | --- | --- | --- | --- |
| 1 | 2 | 3 | 4 | 5 | 6 | 7 |

1. How **dizzy** were you feeling just before you received the trigger?

| Not dizzy at all |  | A little dizzy |  | Quite dizzy |  | Very dizzy |
| --- | --- | --- | --- | --- | --- | --- |
| 1 | 2 | 3 | 4 | 5 | 6 | 7 |

1. How **stiff** were you just before you received the trigger?

| Not stiff at all |  | A little stiff |  | Quite stiff |  | Very stiff |
| --- | --- | --- | --- | --- | --- | --- |
| 1 | 2 | 3 | 4 | 5 | 6 | 7 |

1. How **short of breath** were you just before you received the trigger?

| Not short of breath at all |  | A little short of breath |  | Quite short of breath |  | Very short of breath |
| --- | --- | --- | --- | --- | --- | --- |
| 1 | 2 | 3 | 4 | 5 | 6 | 7 |

1. In the next two hours, I **can** move for at least 10 minutes.

| Strongly disagree |  | A little agree |  | Agree |  | Strongly agree |
| --- | --- | --- | --- | --- | --- | --- |
| 1 | 2 | 3 | 4 | 5 | 6 | 7 |

1. In the next two hours, I **will** move for at least 10 minutes

| Strongly disagree |  | A little agree |  | Agree |  | Strongly agree |
| --- | --- | --- | --- | --- | --- | --- |
| 1 | 2 | 3 | 4 | 5 | 6 | 7 |
