# Supplementary material for: Within-Person Associations of Accelerometer-Assessed Physical Activity With Time-Varying Determinants in Older Adults: Time-Based Ecological Momentary Assessment Study
Source: JMIR Aging. 2023 Nov 23;6:e44425. doi: 10.2196/44425 (PMC10704312; doi:10.2196/44425)
Supplement: Multimedia Appendix 2 [file aging_v6i1e44425_app2.docx]

## Appendix 2

### Descriptive statistics of the TPA data.

|  |  | **M ± SD** | **Range** | **median** | **Q1 – Q3** | **% of pp who did not perform any PA** |
| --- | --- | --- | --- | --- | --- | --- |
| **TPA** | **15 min** | 2.4 ± 3.6 | 0.0 – 15.0 | 0.0 | 0.0 – 4.0 | 51.0 |
|  | **30 min** | 4.7 ± 6.4 | 0.0 – 30.0 | 2.0 | 0.0 – 7.0 | 37.1 |
|  | **60 min** | 9.2 ± 11.3 | 0.0 – 57.0 | 5.0 | 1.0 – 13.0 | 21.6 |
|  | **120 min** | 17.5 ± 19.4 | 0.0 – 105.0 | 10.0 | 3.0 – 26.0 | 9.6 |
