# Supplementary material for: Within-Person Associations of Accelerometer-Assessed Physical Activity With Time-Varying Determinants in Older Adults: Time-Based Ecological Momentary Assessment Study
Source: JMIR Aging. 2023 Nov 23;6:e44425. doi: 10.2196/44425 (PMC10704312; doi:10.2196/44425)
Supplement: Multimedia Appendix 3 [file aging_v6i1e44425_app3.docx]

## Appendix 3

### Outcomes of the logistic models and negative binomial models for TPA.

|  | | | **Logistic model** | | **Neg. binomial** | |  |
| --- | --- | --- | --- | --- | --- | --- | --- |
|  |  |  | **OR (95% CI)** | **p-value** | **expB (95% CI)** | **p-value** |  |
| **Relaxation** | TPA | 15 min | 0.92 (0.84 – 1.00) | .04 | 0.98 (0.94 – 1.02) | .42 |  |
|  |  | 30 min | 0.90 (0.82 – 0.98) | .02 | 0.97 (0.93 – 1.01) | .17 |  |
|  |  | 60 min | 0.90 (0.81 – 1.00) | .04 | 0.95 (0.91 – 0.98) | .005 |  |
|  |  | 120 min | 0.77 (0.65 – 0.91) | .002 | 0.94 (0.90 – 0.97) | < .001 |  |
| **Satisfaction** | TPA | 15 min | 0.91 (0.83 – 1.00) | .04 | 1.00 (0.96 – 1.04) | .998 |  |
|  |  | 30 min | 0.91 (0.83 – 1.01) | .07 | 0.98 (0.94 – 1.02) | .35 |  |
|  |  | 60 min | 0.93 (0.84 – 1.04) | .22 | 0.97 (0.93 – 1.01) | .13 |  |
|  |  | 120 min | 0.89 (0.76 – 1.05) | .18 | 0.95 (0.92 – 0.99) | .02 |  |
| **Irritation** | TPA | 15 min | 1.02 (0.91 – 1.13) | .78 | 0.98 (0.92 – 1.03) | .39 |  |
|  |  | 30 min | 1.14 (1.01 – 1.28) | .04 | 0.97 (0.92 – 1.02) | .23 |  |
|  |  | 60 min | 1.15 (1.00 – 1.33) | .05 | 1.02 (0.96 – 1.07) | .57 |  |
|  |  | 120 min | 1.27 (1.02 – 1.58) | .03 | 1.05 (1.00 – 1.10) | .04 |  |
| **Feeling down** | TPA | 15 min | 1.22 (1.06 – 1.41) | .005 | 0.96 (0.90 – 1.02) | .17 |  |
|  |  | 30 min | 1.18 (1.02 – 1.38) | .03 | 1.02 (0.95 – 1.08) | .61 |  |
|  |  | 60 min | 1.22 (1.01 – 1.46) | .04 | 1.03 (0.97 – 1.10) | .35 |  |
|  |  | 120 min | 1.50 (1.08 – 2.08) | .02 | 1.05 (0.99 – 1.12) | .08 |  |
| **Fatigue** | TPA | 15 min | 0.94 (0.85 – 1.05) | .26 | 0.95 (0.90 – 1.00) | .06 |  |
|  |  | 30 min | 0.92 (0.82 – 1.02) | .13 | 0.93 (0.88 – 0.98) | .01 |  |
|  |  | 60 min | 0.81 (0.72 – 0.92) | .001 | 0.90 (0.85 – 0.94) | < .001 |  |
|  |  | 120 min | 0.73 (0.60 – 0.88) | .001 | 0.87 (0.83 – 0.91) | < .001 |  |
| **Intention** | TPA | 15 min | 1.33 (1.26 – 1.41) | < .001 | 1.12 (1.09 – 1.15) | < .001 |  |
|  |  | 30 min | 1.40 (1.32 – 1.50) | < .001 | 1.16 (1.13 – 1.19) | < .001 |  |
|  |  | 60 min | 1.45 (1.34 – 1.57) | < .001 | 1.23 (1.20 – 1.26) | < .001 |  |
|  |  | 120 min | 1.45 (1.28 – 1.64) | < .001 | 1.28 (1.26 – 1.31) | < .001 |  |
| **Self-efficacy** | TPA | 15 min | 1.27 (1.19 – 1.35) | < .001 | 1.09 (1.06 – 1.12) | < .001 |  |
|  |  | 30 min | 1.32 (1.23 – 1.41) | < .001 | 1.13 (1.10 – 1.16) | < .001 |  |
|  |  | 60 min | 1.38 (1.26 – 1.49) | < .001 | 1.19 (1.16 – 1.23) | < .001 |  |
|  |  | 120 min | 1.37 (1.21 – 1.57) | < .001 | 1.21 (1.18 – 1.24) | < .001 |  |
| *OR = odds ratio ; CI = confidence interval* | | | | | | | |
