# Supplementary material for: Within-Person Associations of Accelerometer-Assessed Physical Activity With Time-Varying Determinants in Older Adults: Time-Based Ecological Momentary Assessment Study
Source: JMIR Aging. 2023 Nov 23;6:e44425. doi: 10.2196/44425 (PMC10704312; doi:10.2196/44425)
Supplement: Multimedia Appendix 5 [file aging_v6i1e44425_app5.docx]

## Appendix 5

Overview of the associations of the determinants with light physical activity (LPA) moderate to vigorous physical activity (MVPA) in the 15, 30, 60 and 120 minutes after the trigger.

|  | **Logistic model** | | | | | | | | **Negative binomial model** | | | | | | | |
| --- | --- | --- | --- | --- | --- | --- | --- | --- | --- | --- | --- | --- | --- | --- | --- | --- |
|  | **LPA** | | | | **MVPA** | | | | **LPA** | | | | **MVPA** | | | |
|  | 15 minutes | 30 minutes | 60 minutes | 120 minutes | 15 minutes | 30 minutes | 60 minutes | 120 minutes | 15 minutes | 30 minutes | 60 minutes | 120 minutes | 15 minutes | 30 minutes | 60 minutes | 120 minutes |
| **Relaxation** |  |  |  |  |  |  |  |  |  |  |  |  |  |  |  |  |
| **Satisfaction** |  |  |  |  |  |  |  |  |  |  |  |  |  |  |  |  |
| **Irritation** |  |  |  |  |  |  |  |  |  |  |  |  |  |  |  |  |
| **Feeling down** |  |  |  |  |  |  |  |  |  |  |  |  |  |  |  |  |
| **Fatigue** |  |  |  |  |  |  |  |  |  |  |  |  |  |  |  |  |
| **Intention** |  |  |  |  |  |  |  |  |  |  |  |  |  |  |  |  |
| **Self-efficacy** |  |  |  |  |  |  |  |  |  |  |  |  |  |  |  |  |
| *Grey = no significant association (P>.05); red = negative significant association (P<.05); green = positive significant association (P<.05)* | | | | | | | | | | | | | | | | |
